# Vaginal Fibroblastic Cells from Women with Pelvic Organ Prolapse Produce Matrices with Increased Stiffness and Collagen Content

**DOI:** 10.1038/srep22971

**Published:** 2016-03-11

**Authors:** Alejandra M. Ruiz-Zapata, Manon H. Kerkhof, Samaneh Ghazanfari, Behrouz Zandieh-Doulabi, Reinout Stoop, Theo H. Smit, Marco N. Helder

**Affiliations:** 1 Department of Orthopedic Surgery, VU University medical center, Research Institute MOVE, Netherlands Institute for Regenerative Medicine, Amsterdam, The Netherlands; 2 Department of Oral Cell Biology, ACTA- University of Amsterdam and VU University, Research Institute MOVE, Amsterdam, The Netherlands; 3 TNO Metabolic Health Research, Leiden, The Netherlands

## Abstract

Pelvic organ prolapse (POP) is characterised by the weakening of the pelvic floor support tissues, and often by subsequent prolapse of the bladder outside the body, *i.e.* cystocele. The bladder is kept in place by the anterior vaginal wall which consists of a dense extracellular matrix rich in collagen content that is maintained and remodelled by fibroblastic cells, *i.e*. fibroblasts and myofibroblasts. Since altered matrix production influences tissue quality, and myofibroblasts are involved in normal and pathological soft tissue repair processes, we evaluated matrix production of cells derived from pre- and post-menopausal POP and non-POP control anterior vaginal wall tissues. Results showed that cells from postmenopausal POP women deposited matrices with high percentage of collagen fibres with less anisotropic orientation and increased stiffness than those produced by controls. There was a transient increase in myofibroblastic phenotype that was lost after the peak of tissue remodelling. In conclusion, affected fibroblasts from postmenopausal prolapsed tissues produced altered matrices *in vitro* compared to controls. Such aberrant altered matrix production does not appear to be a consequence of abnormal phenotypical changes towards the myofibroblastic lineage.

Pelvic organ prolapse (POP) is characterised by the weakening of the pelvic floor supportive tissues, with subsequent prolapse of the uterus, rectum and/or most commonly the bladder outside the body^1^,^2^,^3^. POP affects almost 50% of postmenopausal women causing them to suffer chronic pain, urinary incontinence, and voiding and sexual dysfunctions^1^,^2^. There is a strong genetic component in some women affected with prolapse^4^ but environmental factors related to increased abdominal pressure, such as parity, physiological ageing and obesity, also seem to play an important role in the development of the disease in many patients^5^.

In healthy women the bladder is kept in place by the connective-tissue layer of the anterior vaginal wall which is a dense extracellular matrix (ECM) with relatively few cells. The ECM obtains its strength from the fibrillar proteins (collagen I, III, V and elastin)^6^ and is produced and maintained by fibroblastic cells, *i.e.* fibroblasts and myofibroblasts. Fibroblastic cells remodel their surrounding matrix and maintain tissue homeostasis by producing anabolic molecules and catabolic enzymes such as the matrix metalloproteinases (MMPs). Matrix production and remodelling affect the composition and mechanical properties of the surrounding tissues whose integrity depends on a balance between ECM synthesis and degradation.

In prolapsed tissues however, this balance seems to be lost as studies indicate that the metabolism of collagen and elastin is altered^7^. In patients with cystocele, the prolapsed anterior vaginal wall tissues were shown to have: disorganized collagen and elastin fibres^8^; increased enzymatic activity^9^,^10^,^11^; altered elastin^12^,^13^ and collagen content^9^,^10^,^14^,^15^,^16^; altered collagen cross-linking^9^,^17^; and increased stiffness^16^,^18^,^19^,^20^. Fibroblastic cells derived from prolapsed anterior vaginal wall tissues are also affected as their contractile capacities are lower than non-prolapsed cells^21^,^22^,^23^, their mechano-responses are altered^23^,^24^, and they seem to have lower responses to transforming growth factor-β (TGF-β)^25^, a recognized activator of myofibroblast differentiation. Prolapse therefore affects tissue composition, tissue mechanical properties and cell behaviour. Nevertheless it is not known if prolapse also affects the capacity of the cells to produce and remodel the ECM. Moreover, fibrillar collagens are the main components of the vaginal ECM, they provide strength to the tissues^6^, and they are involved in fibroblast to myofibroblast differentiation^26^, which is a process involved in healthy and pathological soft tissue repair and remodelling and has not been studied in the context of POP. In the present study we hypothesized that prolapse affects fibroblastic cells collagen matrix production and remodelling. To test our hypothesis we evaluated the *in vitro* matrix production of primary cells derived from vaginal tissues from pre- and post-menopausal women with and without POP. The quantity and quality of the deposited matrix were tested for total protein content, total fibrillar collagen content and collagen cross-linking. In particular, collagen I fibre orientation was visualized and quantified. The mechanical properties of the matrices were measured using micro-indentation. We also followed cellular differentiation towards the myofibroblastic phenotype using α-smooth muscle actin (α-SMA) which was detected by western immunoblotting.

## Results

### Anterior vaginal wall cells from patients with prolapse deposit extracellular matrices with less protein content than control cells, but with high collagen content

**Figure 1:**
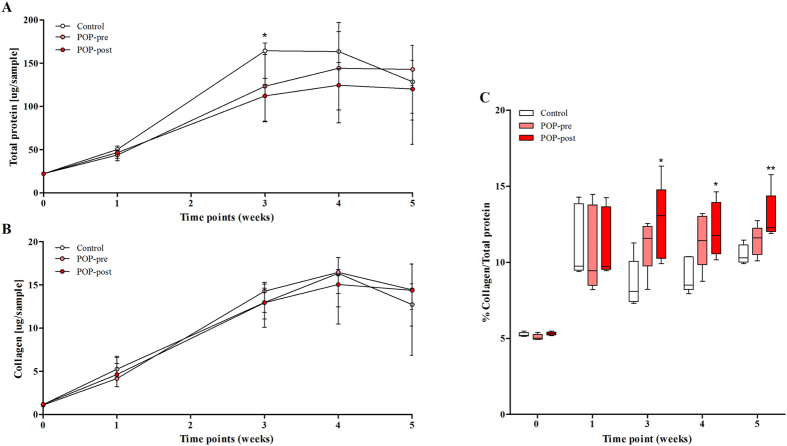
Fibroblastic cells from postmenopausal prolapsed tissues deposit less extracellular matrix than controls, but with high collagen content. Cells derived from prolapsed tissues from premenopausal (POP-pre) and postmenopausal (POP-post) women were cultured *in vitro* for five weeks in the presence of vitamin C and compared to controls. The deposited matrices were stained with Sirius red/fast green collagen staining kit (Chondrex Inc.) at different time points. Data represent the median (+/− interquartile range) of five samples per group and is shown as: (**A**) total protein (control vs. POP-post: p = 0.0486), (**B**) collagen content, and (**C**) box plots of the percentage (%) of collagen content per total protein (control vs. POP-post at week 3, p = 0.0327; at week 4, p = 0.0400; and week 5, p = 0.0089). Differences between control and POP-post were identified by Kruskal-Wallis test followed by Dunn’s multiple comparison: *p < 0.05 and **p < 0.01.

The primary aim of this study was to find out if cells isolated from anterior vaginal wall tissues from women with cystocele deposit different extracellular matrices (ECM) *in vitro* than cells derived from controls (non-prolapsed tissues). The cells were cultured for five weeks in the presence of vitamin C, and matrix production was tested at 0, 1, 3, 4 and 5 weeks. Results show that matrix production from vaginal cells derived from prolapsed tissues was lower than those from controls, however with high collagen content. Matrix deposition increased overtime with peak values at week 3 for controls, and at week 4 for POP matrices (Fig. 1A). The total amount of protein was lower in POP than in control matrices only at week 3 (Fig. 1A) but the collagen content was similar (Fig. 1B). The percentage of collagen fibres in matrices produced by cells from postmenopausal women with prolapse was higher than in the matrices deposited by cells from controls (Fig. 1C). The same trend was seen in matrices from premenopausal women with POP (Fig. 1C).

### Fibroblastic cells from women with prolapse produced stiffer matrices than controls

Pelvic organ prolapse has been shown to affect cell behaviour *in vitro*^21^,^22^,^23^,^24^. To investigate if prolapse also affects the quality of the matrix produced by vaginal cells we tested the surface micro-stiffness of matrices deposited after 5 weeks of culture. We tested the matrices using a micro-indentor with a soft cantilever on top of a ferruled optical fibre. Results showed that fibroblastic cells derived from postmenopausal women produced stiffer matrices than cells from non-prolapsed tissues with the effective Young modulus being 3 times higher. The same trend was seen in matrices deposited by cells from premenopausal women with prolapse (Table 1).

### Collagen type I fibres and cell nuclei were aligned in one preferential direction in control and premenopausal POP groups but not in the postmenopausal POP group

**Figure 2:**
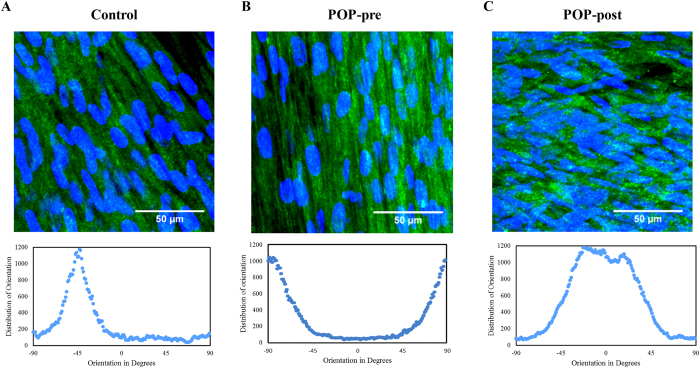
Collagen type I fibres and cell nuclei were aligned in one preferential direction in control and premenopausal POP groups but not in the postmenopausal POP group. Representative immunohistochemistry micrographs of collagen I (green) and cells nuclei (blue) in deposited matrices after five weeks of culture of cells derived from (**A**) control, (**B**) prolapsed premenopausal (POP-pre), and (**C**) prolapsed postmenopausal (POP-post). The figure depicts the maximum intensity projection from z-stacks (top panel) and their corresponding fiber orientation (bottom panel). Images were acquired with 63 x objective of a Leica microscope. The bar is 50 μm.

One of the main components of the ECM in the anterior vaginal wall is collagen I. Since the orientation of the collagen fibres influences the mechanical properties of the ECM^27^ we visualised the collagen I fibre alignment in the deposited matrices after five weeks of culture. Collagen type I fibres and cell nuclei were anisotropically aligned towards a preferential but unspecific direction in all matrices derived from control and premenopausal prolapsed cells (Fig. 2A,B). In contrast, 83% of the matrices derived from prolapsed postmenopausal cells had collagen fibres which appeared to have two anisotropically oriented layers (Fig. 2C).

### The mature collagen cross-links hydroxylysylpyridinoline (HP) and lysylpyridinoline (LP) were similar

Collagen molecules in POP tissues have been reported to have more mature cross-links than those from non-POP tissues^9^,^17^ and we wanted to investigate if this was the case in matrices deposited by vaginal cells *in vitro*. The high-performance liquid chromatography (HPLC) technique which was used can detect only the mature collagen cross-links hydroxylysylpyridinoline (HP) and lysylpyridinoline (LP). After five weeks of culture, cells were freeze-thawed three times and post-incubated for an additional three weeks in PBS at 37 °C to allow the development of mature cross-links. No differences were seen in the amount of collagen cross-links within the deposited matrices (Table 2).

### Proliferation of primary human anterior vaginal wall cells was not affected by prolapse nor by menopausal status

**Figure 3:**
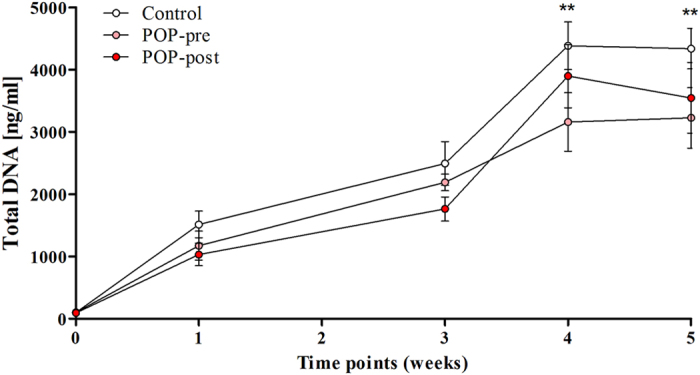
Proliferation of anterior vaginal wall fibroblastic cells was not affected by POP or menopausal status *in vitro*. The total DNA of fibroblastic cells was measured by CyQuant at different time points. The cells were derived from prolapsed tissues from premenopausal (POP-pre) and postmenopausal (POP-post) women, as well as from controls. The box plots correspond to five samples per group per time point. **p < 0.01 by Kruskal-Wallis test followed by Dunn’s multiple comparison compared to week 0. Week 0 vs. week 4: control, p = 0.0019; POP-pre, p = 0.0069; POP-post, p = 0.0011. Week 0 vs. week 5: control, p = 0.0019; POP-pre, p = 0.0031; POP-post, p = 0.0031. No differences were found between the study groups.

The quality and quantity of the deposited matrices can be affected by the type of cell, the number of cells and by their proliferation rate. We first characterised the initial population of primary fibroblastic cells by immunocytochemistry, and we confirmed that the studied cells were from the mesenchymal lineage (vimentin positive), at least 95% smooth muscle free (desmin negative), and negative for the endothelial cell marker Ulex Europaeus Agglutinin-I (UEA-1). These results were consistent with our previous report^23^. We also measured the total DNA of each cell type at the different time points during the five weeks of culture. Cells proliferated normally with a peak at week 4, and with almost no differences in cell number between weeks 4 and 5 (Fig. 3). We found no differences in the proliferation rates of the studied groups.

### Vaginal fibroblastic cells secreted matrix metalloproteinases-2 mainly in the active form and with peak secretion at week three

**Figure 4:**
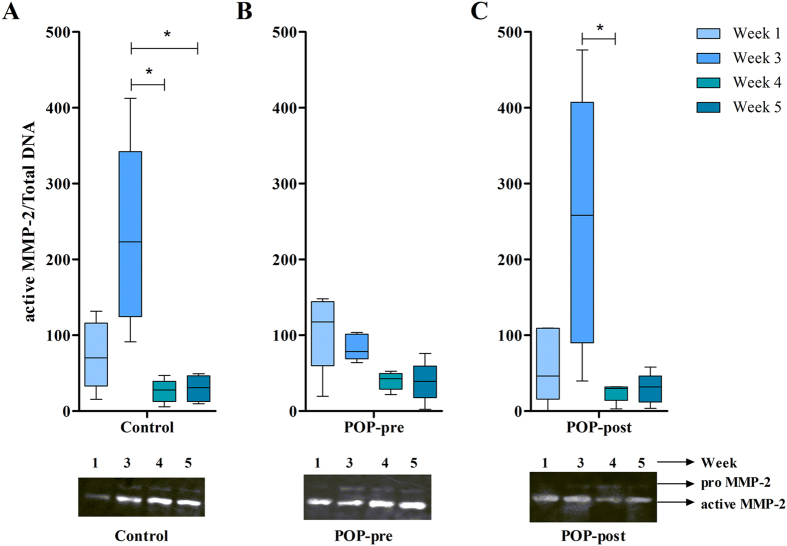
Vaginal fibroblastic cells secreted and activated matrix metalloproteinases-2 in long term cultures *in vitro.* Conditioned media of fibroblastic cells from prolapsed premenopausal (POP-pre) (**B**) and postmenopausal (POP-post) **(C)** tissues was evaluated at different time points and compared to controls (**A**). Matrix metalloproteinase (MMP)-2 was detected by zymography, in samples derived from the same experiment, with gels processed in parallel. The blots were quantified by densiometry analysis of the bands and data were normalised to total DNA. The box plots correspond to five samples per group per time point. *p < 0.05 by Kruskal-Wallis test followed by Dunn’s multiple comparison. Week 3 vs. week 4: control, p = 0.0116; and POP-post, p = 0.0234. No differences were found between the study groups. Full-length gels are presented in Supplementary Figure S1.

Tissue remodelling seems to be affected in tissues from patients with POP. To achieve a better understanding of tissue remodelling we detected secreted active, and inactive (pro form) matrix metalloproteinases (MMP) -2 and -9 by zymography. To correct for the difference in cell numbers at the different experimental time points, quantitative data was normalised to total DNA. Zymogram results show that most of the secreted enzymes were in the active form of MMP-2. In general, the amount of active MMP-2 secreted by the cells increased from weeks one to three, except for prolapsed fibroblasts from premenopausal women (Fig. 4B), and decreased again at week four (Fig. 4A,C). No major differences were seen between weeks 4 and 5. At the peak of secretion (week three) cells from postmenopausal women with prolapse released slightly higher amounts of active MMP-2 than control cells. At the same time point, cells from premenopausal women with prolapse secreted lower quantities of active MMP-2 than cells from the other two groups but the difference was not significant (Fig. 4). We were unable to detect MMP-9, and MMP-2 at week 0 was below detection levels (Supplementary Figure S1).

### Altered matrix production by prolapsed fibroblasts was not a consequence of abnormal phenotypical changes towards the myofibroblastic lineage

**Figure 5:**
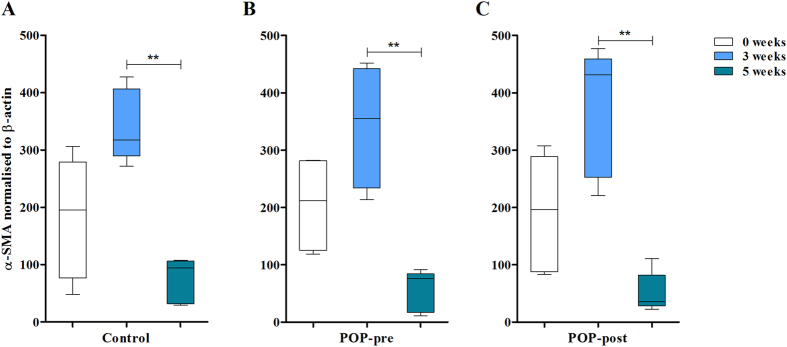
Vaginal fibroblasts show transient differentiation to myofibroblasts *in vitro*. Cell lysates were collected at different time points (0, 3 and 5 weeks) and myofibroblast differentiation was detected by western immunoblotting of α-smooth muscle actin (α-SMA) and β-actin was used as a control. Cells originated from three groups: (**A**) controls, (**B**) prolapsed premenopausal (POP-pre), and (**C**) prolapsed postmenopausal (POP-post). Samples derived from the same experiment were processed in gels in parallel. Blots were quantified by densiometry analysis of the bands and data was normalised to the first band of β-actin per blot. The box plots correspond to five samples per time point per group. **p < 0.01 by Kruskal-Wallis test followed by Dunn’s multiple comparison. Week 3 vs. week 4: control, p = 0.0056; POP-pre, p = 0.0066; POP-post, p = 0.0044 No differences were found between the study groups. Full-length blots are presented in Supplementary Figure S2.

It has been shown *in vitro* that when matrix stiffness is increased fibroblasts differentiate into myofibroblasts^26^. To find out whether the increased substrate stiffness of the prolapsed matrices was the result of a change in phenotype from fibroblasts to myofibroblasts, we detected the myofibroblastic marker α-smooth muscle actin (α-SMA) at the beginning of the experiments (week 0), at the peak of protein deposition (week 3), and at the ending time point (week 5). Results showed a transient increase in myofibroblastic phenotype as α-SMA increased by week three, and thereafter were significantly lower from weeks three to five. There were no differences in cellular α-SMA protein content between the groups (Fig. 5 and Supplementary Figure S2).

## Discussion

Clinical studies have shown that in patients with cystocele, the prolapsed tissues have increased stiffness^16^,^18^,^19^,^20^, altered extracellular matrix composition^8^,^9^,^10^,^11^,^12^,^13^,^14^,^15^,^16^,^17^, and fibroblastic cells with lower contractile capacities^21^,^22^,^23^ and altered mechano-responses^23^,^24^. Our previous studies have shown that some of these differences seem to be acquired in some premenopausal Caucasian women with prolapse^17^,^23^, but the mechanisms behind these conditions are still unclear. Cells are responsible for tissue maintenance and remodelling. If matrix production is altered in prolapsed tissues, treatment outcomes could be improved by understanding the mechanisms behind the altered cell-matrix interactions. Our study has shown that cells from prolapsed anterior vaginal wall tissues produce different extracellular matrices *in vitro* than cells derived from control tissues. The matrices deposited by cells from postmenopausal tissues were stiffer, with a high percentage of collagen, and with collagen I fibres with more than one preferred orientation. These data suggest that matrix production changed after cells had been exposed to prolapsed tissues and that the altered matrix production was more apparent in postmenopausal women. We also found no differences in the number of cells between the groups, thus confirming our previous observations and suggesting that the quality, and not the quantity, of the cells is responsible for the results^23^.

Several *in vivo* studies have shown that prolapsed tissues from women with POP are stiffer than controls^16^,^18^,^19^,^20^, which is in line with our *in vitro* findings. The reason for this increased stiffness *in vivo* remains unclear as results about the composition of the prolapsed matrix have been different and sometimes contradictory^28^,^29^. The amount of collagen cross-links might play an important role as they have a big influence on the mechanical properties of tissues^27^, and they have been reported to be increased in prolapsed tissues^9^,^17^. The values of the mature cross-links that we found in our matrices were below the values that have been reported in tissues^17^, indicating that the formation of mature collagen cross-links in our *in vitro* experiments was still premature. It is also important to note that the formation of mature collagen cross-links might be different *in vivo* from *in vitro*. The increased stiffness that we found in the POP matrices *in vitro* is likely to be a consequence of the high percentage of collagen present and the structural difference found in the collagen I fibres. Matrix production from vaginal cells derived from prolapsed tissues was lower than those from controls, but with a high percentage of collagen fibres. The same trend was seen in matrices from premenopausal women with POP suggesting that the premenopausal status seems to ameliorate, and not reverse the adverse cellular defects in prolapsed tissues. The collagen I fibres found in the deposited matrices had anisotropic orientation that seems to be an intrinsic ordering principle, since no external forces were applied to the system and there were no physical constraining boundaries. This self-organization might be due to the spontaneous self-organization of the high density of collagen, and to the cells’ producing and arranging the fibres within the matrix or a combination of both^27^. The fact that the stiffer matrices derived from postmenopausal prolapsed cells appeared to have collagen I fibres with two anisotropically oriented layers, remains intriguing.

During wound tissue repair, fibroblasts are recruited to the site of injury and they can differentiate into myofibroblasts. When myofibroblasts appear, the remodelling phase starts and the granulose tissue is replaced by a scar tissue rich in collagen I^30^. In this study we used primary fibroblastic cells, *i.e.* fibroblasts and myofibroblasts. Fibroblasts can differentiate into myofibroblasts due to various stimuli including matrix stiffness^26^. When cells produce the proper matrix, tension in their micro-environment can be released and myofibroblasts should disappear either due to apoptosis or to their dedifferention back into fibroblasts^26^. If the mechanical tension of the micro-environment is not released, myofibroblasts might not disappear, making stiffer matrices and causing a continuous imbalance in tissue remodelling, a common feature in fibrotic tissues^26^,^31^. In our experiments we found a transient myofibroblast differentiation with peak expression of the cellular myofibroblast marker α-smooth muscle actin (α-SMA) at week three that coincided with the peak of matrix deposition and with the peak of secretion and activation of matrix metalloproteinase-2 (MMP-2). The peak of activation of MMP-2 found in cells from the controls, and from the postmenopausal POP group, was not evident in cells from premenopausal POP women, even though the maximum matrix deposition and myofibroblast differentiation was still found at week three. Therefore, we speculate that the peak of MMP-2 activation of cells from premenopausal POP women occurred before week three, but this should be further confirmed. Regardless of this variation, it is reasonable to say that the peak of remodelling occurred at week three. These results are in line with normal tissue repair process where myofibroblasts secrete the new matrix, MMPs and tissue inhibitor of matrix metalloproteinases (TIMPs) to remodel the granulose tissue^30^. However, no differences were found between the study groups, and myofibroblasts seemed to disappear after the peak in matrix remodelling. Therefore, the altered matrix production does not seem to be a consequence of abnormal phenotypical changes towards the myofibroblastic lineage.

It has been hypothesised that the weakening of the prolapsed tissues is a consequence of the increased enzymatic activity, particularly of MMP-2 and MMP-9^9^,^10^,^11^. In our *in vitro* experiments we did not find an increase of MMP-2 enzymatic activity in fibroblastic prolapsed cells compared to controls. There are several possible explanations for the discrepancies between *in vivo* and *in vitro* observations of MMP activity: (i) other cells are responsible for the excessive *in vivo* enzymatic activity, (ii) mechanical loading induces MMP secretion and activation^23^,^24^,^32^ (our *in vitro* model was under static conditions), (iii) the differences occurred at time points other than the studied times, (iv) the experiments were performed on substrates which were different to the native micro-environments. In the present study we focused on total fibrillar collagen production and remodelling. However, characterizing the exact composition of the deposited matrices and correlating that information to the mechanical properties would be an interesting topic for a future study involving proteomics.

In summary, affected fibroblasts from postmenopausal prolapsed tissues produced altered matrices *in vitro* compared to controls. Similarly to normal wound repair, there was a transient increase in myofibroblastic phenotype that was lost after the peak of tissue remodelling. The altered matrix production in prolapsed tissues does not appear to be a consequence of abnormal phenotypical changes towards the myofibroblastic lineage.

## Methods

### Patient selection, tissue processing, and cell isolation

Patients were recruited from three hospitals in the Netherlands: Kennemer Gasthuis Hospital in Haarlem, VU University Medical Centre, and the Boerhaave Medical Centre in Amsterdam. After informed consent was obtained from all patients and according to institutional guidelines, biopsies were collected from three groups of Caucasian women. A control group of five women undergoing hysterectomy for benign gynaecological reasons, and two groups of women undergoing prolapse surgery (POP-Q stages ≥ II): five premenopausal women (POP-pre), and five postmenopausal women (POP-post). Women were non-smokers with similar parity and similar POP-Q cystocele stages in POP cases (Table 3). Exclusion criteria were previously described^17^. The tissues collected in the present study were waste material after surgery and not subject to Ethical Committee approval. Full thickness (1 cm^2^) vaginal wall tissue biopsies were taken from the pericervical region of the anterior vaginal cuff after hysterectomy in the controls, and from the prolapsed vaginal wall in the POP cases. A total of 15 different vaginal primary fibroblastic cells were isolated within 24 hours of tissue extraction and cultured using enzymatic digestion as previously described^24^. Cells were grown in an incubator at 37 °C, 95% humidity and 5% CO_2_, with culture medium: Dulbecco’s modified Eagle’s medium-DMEM (Gibco-Life technologies, Paisley, UK) supplemented with 10% foetal bovine serum (FBS; HyClone, South Logan, UT, USA), 100 μg/ml streptomycin, 100 U/ml penicillin, and 250 μg/ml amphotericin-B (Sigma-Aldrich, St. Louis, MO, USA). The cells used in this study were from passages two to four and were at least 94% viable. The initial population of primary cells was characterized by immunocytochemistry analysis as previously described^23^, with markers for mesenchymal cells (vimentin; DakoCytomation, Copenhagen, Denmark), smooth muscle cells (Desmin; DakoCytomation), and endothelial cells (Ulex Europaeus Agglutinin I; Vector Laboratories, Burlingame, CA, USA).

### *In vitro* matrix production

The extracellular matrix produced over five weeks by human vaginal primary cells was evaluated for different outcome parameters *in vitro*. Following the manufacturer’s instructions, cells were counted using the Muse® count & viability assay kit with the Muse® cell analyzer system (Merck Millipore, Darmstadt, Germany). Cells were cultured with culture media and a seeding density of 10,000 cells/cm^2^ in plastic tissue-culture plates and analysed at different time points: 24 hours (0W), 1 weeks (1W), 3 weeks (3W) and 5 weeks (5W). After 24 hours, cells were synchronized for 1 hour at 5 °C, and 0W plates were processed as follows: to detect released matrix metalloproteinases (MMPs)-2 and -9, media was replaced by culture media containing 1% FBS (HyClone) and post-incubated for 6 hours in an incubator at 37 °C, 95% humidity and 5% CO_2_. Conditioned media was saved for zymogram analysis and cell lysates were collected for total DNA analysis. Samples were also prepared for total fibrillar collagen content.

The rest of the cultures were refreshed with culture media supplemented with 50 μg/ml vitamin C (Sigma-Aldrich), and thereafter refreshed every 3 to 4 days with the same medium. After each time point, samples were processed as at 0W. Additionally, samples were collected at 0W, 3W, and 5W for western immunoblotting. At 5W, samples were also collected for surface micro-stiffness, collagen I immunostaining and biochemical analysis (see relevant sections below).

### Zymogram analysis of secreted MMP-2 and MMP-9

Secreted MMP-2 and MMP-9 were detected by zymography following the manufacturer’s protocol for Novex zymogram gels (10% zymogram gelatin gel, Life Technologies). The dark bands of gelatinolytic activity were visualized with the eStain protein staining device (GeneScript, Piscataway, NJ, USA). Images were acquired with the Biospectrum AC Imaging System (UVP, Cambridge, UK) and zymogram quantification of the density of the bands was performed using Image J 1.44p software (National Institutes of Health, USA).

### Total DNA analysis

Fibroblastic cells were seeded on 48-well plates and samples were collected at different time points: 0, 1, 3, 4 and 5 weeks. At the end of each time point, cells were washed with PBS, and 300 μl/well of milliQ water was added before samples were stored at −20 °C until further analysis. After three freeze and thaw cycles, total DNA of duplicate samples was measured with the CyQuant cell proliferation assay kit according to the manufacturer’s specifications (Molecular Probes Inc., Life Technologies). Fluorescence was measured using the Synergy^TM^HT multi-mode microplate reader (Biotek Instruments Inc.).

### Detection and quantification of fibrillar collagens and non-collagenous proteins

The total protein content, and the fibrillar collagen content of the deposited matrices, was measured with the Sirius red/fast green collagen staining kit (Chondrex Inc., Redmond, USA) following the manufacturer’s specification. The optical density values (OD) of eluted dye were measured at 540 nm and 605 nm with the Synergy^TM^HT multi-mode microplate reader (Biotek Instruments Inc.). Calculations were made with the formulas: total protein = {[OD_540_ −  (OD_605_ × 0.291)]/0.0378} + OD_605_/0.00204; collagen = [OD_540_ − (OD_605_ × 0.291)]/0.0378.

### Western immunoblotting

Cells cultured on 6-well plates were lysed with 700 μl/well of Lysis-M buffer including proteinase inhibitor (Roche Diagnostics GmbH, Mannheim, Germany). Protein content was determined using the Pierce BCA Protein Assay kit following the manufacturer’s instructions (Thermo Scientific, Rockford, USA). Equal amounts of protein were denatured and separated by electrophoresis on a Bolt® 4–12% Bis-Tris Plus gel under reducing conditions and transferred to iBlot® PVDF membrane (Life Technologies). Blots were blocked for 1 hour at room temperature with a blocking buffer (PBS with 0.5% Tween-20 and 1% bovine serum albumin; Sigma-Aldrich), then incubated with anti-α-SMA monoclonal antibody (clone 1A4, DakoCytomation) diluted 1:500 in blocking buffer for 1 hour at room temperature and subsequently overnight at 4 °C. Bound antibodies were visualized with a horseradish peroxidase-conjugated antibody goat anti-mouse (1:10,000) and the Supersignal west pico chemiluminescence kit (Thermo Scientific). Images were acquired with the Biospectrum AC Imaging System (UVP). Gamma settings were optimized for band clarity. After detection, blots were washed with blocking buffer, incubated for 1 hour at room temperature with mouse anti-β-actin antibody (1:5,000; Sigma-Aldrich), and washed three times with PBS. To visualize the bound antibodies with alkaline phosphatase (AP) chromogenic detection blots were washed for 10 minutes at room temperature with Tris Buffer Saline pH 7 (TBS: 0.1M tris with 0.9% NaCl; Sigma-Aldrich), then washed for 10 minutes with AP buffer pH 9.5–10 (100 mM NaCl, 0.1 M HCl and 5 mM MgCl_2_; Sigma-Aldrich), and detected with NBT/BCIP substrate (Roche diagnostics GmbH) diluted in AP buffer (1:100). Images were acquired with the Biospectrum AC Imaging System (UVP). Quantification of the band density was performed using Image J 1.44p software (NIH).

### Surface micro-stiffness

The mechanical properties of the deposited matrices were measured using a newly-developed nano-indentor designed to test the surface stiffness of biological tissues (PIUMA, Optics11, Amsterdam, The Netherlands). After five weeks of culture two fresh samples were indented in PBS with the PIUMA device and according to supplier’s instructions (Optics11). We used a soft cantilever on the top of a ferruled optical fibre^33^,^34^ with a radius of 88 μm and a stiffness of 0.85 N/m. The indentations were depth controlled (15 μm) and the loading and unloading periods were set to 3 seconds. Based on the load and displacement curves, the effective Young’s modulus was automatically calculated by the software from the PIUMA (Optics11). To calibrate the probe and the measurement technique, a stiff surface was used before each series of tests.

### Collagen I immunostaining

Matrices deposited after five weeks of culture were fixated using 4% formaldehyde, then incubated with blocking buffer (1% BSA, 20% donkey serum, PBS) for 1 hour followed by 2 hours incubation with rabbit polyclonal anti-collagen type I antibody (1:1000, Abcam, USA) at room temperature. After three washings with PBS, Alexa 488 donkey anti-rabbit (1:2000, Invitrogen, USA) was added for 1 hour. Then the slides were washed three times in PBS and mounted with Vectashield mounting medium containing DAPI (Vector Laboratories, USA). Images were taken using an inverted confocal microscope (Leica SP8, Leica, Germany). To evaluate the local fibre orientation we used Orientation J which is an Image J plug-in (NIH) and calculates the local orientation of fibres in every pixel of the image.

### Biochemical analysis of the collagen cross-links hydroxylysylpyridinoline (HP) and lysylpyridinoline (LP)

Cells were cultured for five weeks on 6-well plates and then freezed-thawed three times. To allow time for the maturation of collagen cross-links, the acellular matrices were post-incubated for three more weeks in PBS at 37 °C. Collagen and the mature collagen cross-links hydroxylysylpyridinoline (HP) and lysylpyridinoline (LP) were measured biochemically with a HPLC system equipped with online sample purification on CC31 cellulose using a Prospekt solid-phase extractor (Separations, Jasco Benelux BV, IJsselstein, The Netherlands) as previously described^17^. The mature collagen cross-links were expressed as (HP + LP)/triple helix.

### Statistics

Data are expressed as the median with the interquartile range (IQR) of five independent samples per group. Analyses were performed with Prism software version 6.0 (GraphPad Software Inc., San Diego, CA, USA) using non-parametric Kruskal-Wallis test followed by Dunn’s multiple comparisons for the grouped analysis or Mann-Whitney test for paired analysis. Differences were considered significant at 5% level (p < 0.05).

## Additional Information

**How to cite this article**: Ruiz-Zapata, A. M. *et al.* Vaginal Fibroblastic Cells from Women with Pelvic Organ Prolapse Produce Matrices with Increased Stiffness and Collagen Content. *Sci. Rep.* **6**, 22971; doi: 10.1038/srep22971 (2016).

## Supplementary Material

Supplementary Information

## Figures and Tables

**Table 1:** Surface micro-stiffness: effective Young modulus (kPa).

	A Control (n = 5)	B POP-pre (n = 5)	C POP-post (n = 5)	A–B p-value	A–C p-value	B–C p-value
Young modulus (kPa)	16 (14.9–29.4)	26 (19.1–100.5)	50 (25.7–86.3)	0.3095	0.0317	0.5476

Data is presented as: median (IQR). Non-parametric statistical tests: Mann-Whitney.

**Table 2:** Biochemical analysis of collagen cross-links hydroxylysylpyridinoline (HP) and lysylpyridinoline (LP).

	Control (n = 5)	POP-pre (n = 5)	POP-post (n = 5)
(HP + LP)/triple helix	0.09 (0.08–0.25)	0.09 (0.04–0.10)	0.10 (0.10–0.12)

Data is presented as: median (IQR). Non-parametric statistical tests: Mann-Whitney.

**Table 3:** Patient characteristics.

Characteristic	A Control (n = 5)	B POP-pre (n = 5)	C POP-post (n = 5)	A–B p-value	A–C p-value	B–C p-value
Age^a^	47.0 ± 4.12	44.6 ± 4.93	59.4 ± 9.34	0.528	0.056	0.032
Parity^b^	3 (1–3)	2 (2–3)	2 (2–3)	0.913	0.914	1.000
POP-Q cystocele stage^b^	0 (0)	2 (2)	2 (2–3.5)			

Data is presented as: mean ± SD^a^, or median (IQR)^b^. Non-parametric statistical tests: Mann-Whitney.
