# Supplementary material for: Vaginal Fibroblastic Cells from Women with Pelvic Organ Prolapse Produce Matrices with Increased Stiffness and Collagen Content
Source: Sci Rep. 2016 Mar 11;6:22971. doi: 10.1038/srep22971 (PMC4786799; doi:10.1038/srep22971)
Supplement: Supplementary Information [file srep22971-s1.pdf]

Supplementary Information

Vaginal fibroblastic cells from women with pelvic organ prolapse produce matrices with increased stiffness and collagen content

Alejandra M. Ruiz-Zapata[1*], Manon H. Kerkhof[1], Samaneh Ghazanfari[1], Behrouz Zandieh-Doulabi[2], Reinout Stoop[3], Theo H. Smit[1] and Marco N. Helder[1]

[1]Department of Orthopedic Surgery, VU University medical center, Research Institute MOVE, Netherlands Institute for Regenerative Medicine, Amsterdam, The Netherlands.
[2]Department of Oral Cell Biology, ACTA- University of Amsterdam and VU University, Research Institute MOVE, Amsterdam, The Netherlands. [3]TNO Metabolic Health Research, Leiden, The Netherlands.

[*]Corresponding author:

Alejandra M. Ruiz-Zapata

Present affiliation and address:

Department of Obstetrics and Gynecology, and Department of Urology
Radboud University Medical Center, Geert Grooteplein Zuid 26-28 (route 267, room 1.75), 6525 GA Nijmegen, The Netherlands.
E-mail: Alejandra.RuizZapata@radboudumc.nl

Supplementary Figures

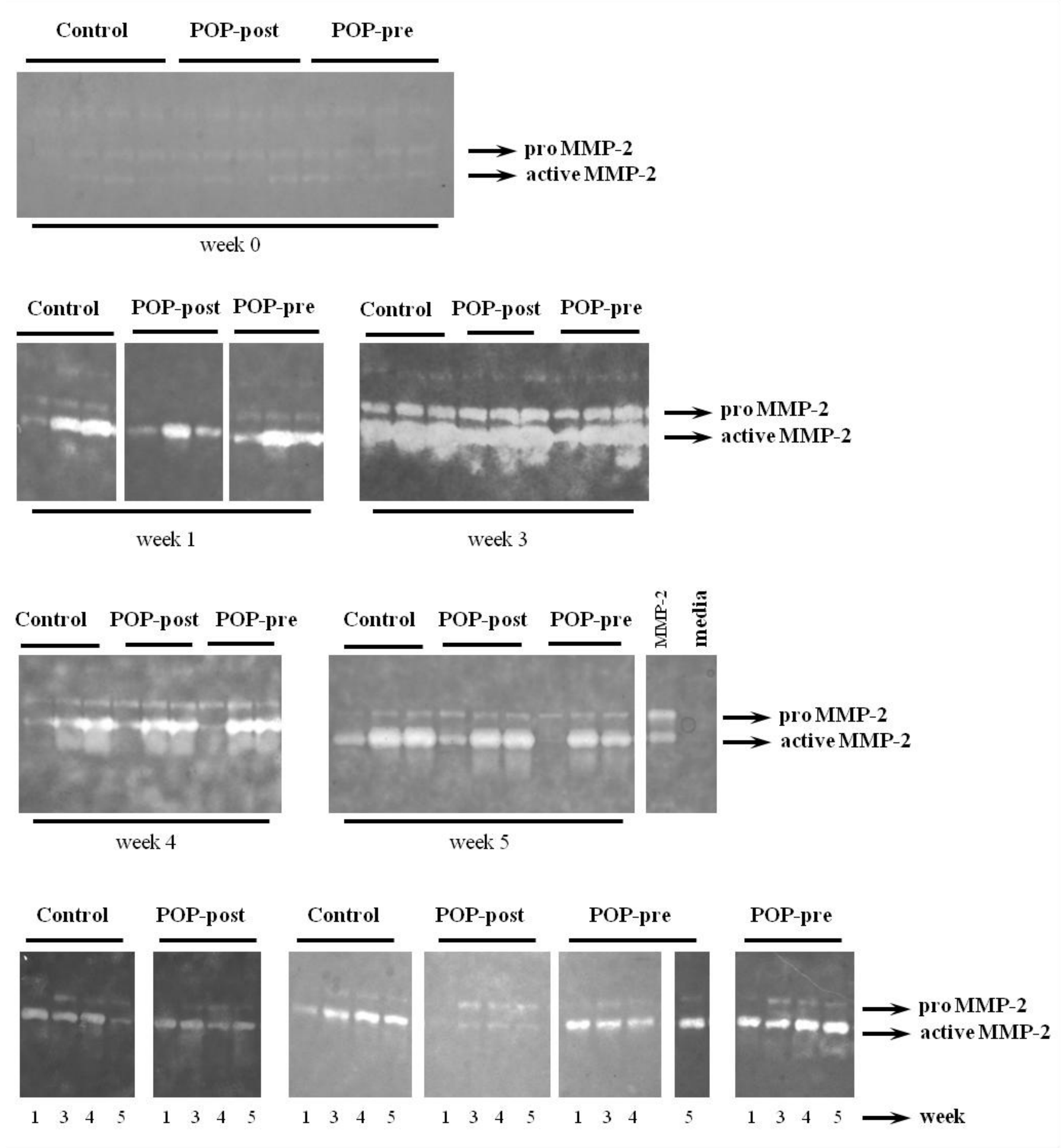

Supplementary Figure S1. Vaginal fibroblastic cells secreted and activated matrix metalloproteinases-2 in long term cultures *in vitro.* Matrix metalloproteinase (MMP)-2 was detected by zymography of conditioned media at different time points of fibroblastic cells from prolapsed premenopausal (POP-pre; n=5), postmenopausal (POP-post; n=5) and healthy control tissues (Control; n=5).

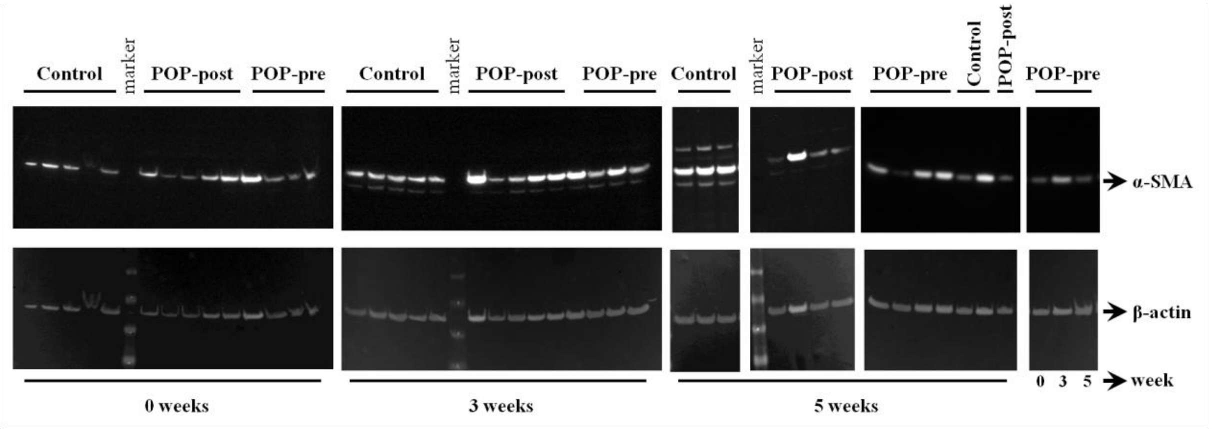

Supplementary Figure S2. Vaginal fibroblasts show transient differentiation to myofibroblasts *in vitro*. Western blots of α-SMA at different time points of cells derived from prolapsed premenopausal (POP-pre; n=5), postmenopausal (POP-post; n=5) and healthy control tissues (Control; n=5).
